# The Pivotal Role of Invasive Functional Assessment in Patients With Myocardial Infarction With Non-Obstructive Coronary Arteries (MINOCA)

**DOI:** 10.3389/fcvm.2021.781485

**Published:** 2021-11-17

**Authors:** Fabio Mangiacapra, Michele Mattia Viscusi, Luca Paolucci, Annunziata Nusca, Rosetta Melfi, Gian Paolo Ussia, Francesco Grigioni

**Affiliations:** Unit of Cardiovascular Science, Department of Medicine, Campus Bio-Medico University, Rome, Italy

**Keywords:** MINOCA, coronary artery disease, acute coronary syndrome, coronary physiology, microvascular dysfunction

## Abstract

Myocardial infarction with non-obstructive coronary arteries (MINOCA) encompasses several pathophysiological mechanisms not yet fully understood. Among the latter, vasomotion abnormalities and coronary microvascular dysfunction (CMD) play a major role for both epidemiological and prognostic reasons. Despite current guidelines do not recommend routine physiological assessment of both epicardial and microvascular coronary compartments within the context of an acute myocardial infarction, several recent evidence support the critical role of a comprehensive invasive functional assessment in order to identify the underlying pathophysiological mechanism and consequently to select an appropriate therapeutic strategy. Unfortunately, optimal medical therapy for these patients is not currently established due to the lack of dedicated trials evaluating clinical outcomes of commonly used medications for secondary prevention in MINOCA patients. For this reason, additional research is warranted to provide personalized treatments for patients affected by this puzzling clinical entity.

## Minoca: Definition, Pathogenesis, and Unmet Clinical Needs

Myocardial infarction with non-obstructive coronary arteries (MINOCA) was first formally labeled as a clinical syndrome in an international expert opinion paper only in 2016 (1), though autoptic evidence potentially referring to this condition have been reported since the 1930s (2, 3). The epidemiological burden of MINOCA has been underestimated for decades due to the lack of a standardized definition, however the most recent epidemiological data suggest an overall prevalence of 6% out of all acute myocardial infarctions (AMI) events (4).

The pathophysiological mechanisms underlying MINOCA are multiple and not fully understood, however vasomotion abnormalities and coronary microvascular dysfunction (CMD) play a prominent role for both epidemiological and prognostic reasons. Therefore, similarly to the algorithm formulated in the EAPCI consensus document on ischemia and non-obstructive coronary arteries (INOCA) (5), a comprehensive invasive functional assessment of both epicardial and microvascular coronary circulation is critical to establish the exact pathophysiological mechanisms of MINOCA and, therefore, to select an appropriate etiology-based therapeutic strategy.

The purpose of this review is to briefly summarize the main pathophysiological mechanisms of MINOCA and to elucidate the critical role of a comprehensive invasive functional assessment. We also report on the main currently available etiology-tailored therapies.

### Definition

The growing body of evidence reporting cases of acute myocardial infarction (AMI) with no significant angiographic findings, particularly with the recent development of high-sensitivity troponin assays, as well as the conflicting positions regarding the heterogeneous pathophysiological mechanisms of MINOCA and the absence of standardized guidelines of management, led the European Society of Cardiology (ESC) in 2017 (1) and the American Heart Association (AHA) in 2019 (6) to publish specific statements to address these issues. According to the latest statement by the AHA, three main criteria need to be met to fulfill the diagnosis of MINOCA: (1) AMI criteria as defined by the “Fourth universal definition of myocardial infarction” (7); (2) non-obstructive coronary arteries on angiography (no coronary artery stenosis ≥ 50%); (3) no specific alternative diagnosis for the clinical presentation. With these more stringent criteria, several disorders that were initially considered as potential causes of MINOCA (e.g., heart failure, Takotsubo syndrome, myocarditis, renal failure, sepsis, arrhythmias, hypotension/shock, pulmonary embolism, stroke, adult respiratory distress syndrome [ARDS]) are no longer considered as such. Consequently, only ischemic causes of AMI are now considered in the pathogenesis of MINOCA, thus contributing to simplify the diagnostic work-up.

### Pathogenesis

Destabilization of vulnerable coronary plaques, spontaneous coronary artery dissection (SCAD), coronary microvascular dysfunction (CMD), coronary thromboembolism, and vasospasm are all potential causes of MINOCA and could be targeted by specific diagnostic tools and therapeutic strategies. The possible etiologies of MINOCA according to the pathological substrate (atherosclerotic/non-atherosclerotic), anatomical localization (epicardial/microvascular) and the type of MI is summarized in Table 1. In particular, the pathophysiological mechanisms of coronary plaque disease, CMD and vasomotion abnormalities will be more deeply discussed in the following paragraphs.

**Table 1:** Causes of MINOCA according to pathological substrate, anatomical localization, and type of AMI.

**Causes**		**Pathological substrate**	**Anatomical localization**	**Type of AMI**
Coronary plaque disease	• Rupture • Erosion	Atherosclerotic	Epicardial	1
Coronary microvascular dysfunction	• Microvascular spasm • Structural remodeling	Non-atherosclerotic	Microvascular	2
Coronary vasospasm	• Epicardial vasospasm	Non-atherosclerotic	Epicardial	2
Coronary thromboembolism	• Acquired/inherited coagulopathies • Paradoxical embolism • Non-thrombotic sources (valvular vegetations, cardiac tumors, calcified valves, iatrogenic air emboli)	Non-atherosclerotic	Epicardial/Microvascular	1
Spontaneous Coronary Artery Dissection (SCAD)	• Multiple radiolucent lumen (*type 1 SCAD*) • long diffuse/smooth stenosis (*type 2 SCAD*) • focal/tubular stenosis (*type 3 SCAD*)	Non-atherosclerotic	Epicardial	2

#### Coronary Plaque Disease

Among MINOCA patients, plaque rupture and plaque erosion play a key role as acute triggers of myocardial infarction mainly producing intraluminal thrombosis with potential overlapping of coronary spasm, distal embolization, or a combination of these processes (8, 9). Of note, plaque composition seems to be highly different in these two pathological entities. An abundance of proteoglycan and glycosaminoglycan in the extra-cellular matrix with dominant endothelial cells apoptosis is usually associated with plaque erosion. In contrast, rupture-prone plaques present a thin fibrous cap with interstitial collagen discontinuation, excess of lipid core and inflammatory cells (10, 11). While plaque erosion more frequently leads to AMI via non-occlusive thrombosis causing distal embolization and possibly superimposed spasm, transient occlusive intraluminal thrombosis with prompt and spontaneous thrombolysis is more frequent in plaque rupture (10).

The introduction of coronary intravascular imaging in ordinary interventional practice allowed a more precise evaluation of the incidence of plaque vulnerability in MINOCA patients. Intravascular ultrasound (IVUS)-based studies have shown an incidence of plaque rupture of ~40% in these patients (12, 13). However, no data regarding the incidence of plaque erosion are currently available, because of the lack of analysis performed within the MINOCA population using optical coherence tomography (OCT), which is the only high-resolution coronary intravascular imaging technique capable of detecting plaque erosion (14). Furthermore, intracoronary imaging allowed the identification of a rare cause of coronary plaque disease, the calcified nodule, which has also been reported as an uncommon cause of MINOCA (15). Recently, the autoptic morphometric analysis of consecutive calcified nodules allowed to identify the fragmentation of the necrotic core calcifications caused by mechanical stress with subsequent overlying luminal thrombosis as a plausible potential mechanism of acute coronary thrombosis and sudden cardiac death (16).

#### Coronary Microvascular Dysfunction

The potential epidemiological impact of CMD in women with ischemic heart disease in the absence of flow-limiting epicardial stenoses was proposed two decades ago after the WISE study (17), which showed that abnormal coronary flow reserve (CFR) was observed in approximately half of women with chest pain and non-obstructive coronary arteries. Similar estimates have been subsequently confirmed by other studies suggesting that 43–54% of MINOCA patients experience microvascular spasm (18, 19) and that CMD may be detected in up to 50% of patients with chest discomfort and non-obstructive coronary arteries on invasive coronary angiography (20).

Both endothelium-dependent and non-endothelium-dependent mechanisms participate in the pathogenesis of CMD. Endothelial dysfunction implies a dynamic dysregulation of coronary microvasculature vasoreactivity, which inhibits the physiological flow-mediated vasodilation occurring in conditions of increased myocardial oxygen consumption (21). The latter may even result in paradoxical vasoconstriction, thus determining microvascular spasm. The diagnostic approach to microvascular tone with the most reliable efficacy-safety profile is the acetylcholine test. According to the Coronary Vasomotion Disorders International Study Group (COVADIS) indications, this vasoreactivity test meets the criteria for microvascular vasospasm when it reproduces the symptoms usually experienced by the patients and triggers ischemic ECG changes in the absence of significative epicardial spasm (20). On the other hand, non-endothelium dependent mechanisms relate to structural remodeling of coronary microvasculature due to an increased wall-to-lumen ratio (intimal thickening and perivascular fibrosis) and a loss of myocardial capillary density (capillary rarefaction), which result in increased microvascular resistances and permanent decline of coronary flow through the microvasculature (22).

In the acute setting of MINOCA, CMD develops as a consequence of two separate mechanisms: a chronic microvascular dysfunction causing prolonged ischemia with a superimposed acute trigger potentially driving to myocardial necrosis or an isolated, acute, hyper-adrenergic tone with prompt and sudden rise of microvascular resistance that may lead to cardiomyocyte death.

#### Coronary Vasospasm

Epicardial vasospasm shares with the microvascular counterpart the identical mechanism related to smooth muscle cells hyperreactivity to both endogenous (e.g., acetylcholine) and exogenous (e.g., cocaine, methamphetamines, fluorouracil) agents (23). According to the COVADIS definition, coronary epicardial vasospasm may be diagnosed if the administration of high-dose intracoronary acetylcholine boluses (20–100 μg) reproduces the symptoms usually experienced by the patients and triggers ischemic ECG changes with significative epicardial spasm (>90% diameter reduction) (20). Epidemiologically, epicardial vasospasm is one the most frequent mechanisms of MINOCA. Its prevalence ranges between 3 and 95%, with significant variations based on ethnicity (24), showing a particular predilection for Asian rather than white patients (25). However, more recent investigations found epicardial vasospasm in 46% of MINOCA patients undergoing acetylcholine provocative tests (26). In addition, a recent contribution by Montone et al. (27) assessed the relationship between myocardial bridging and coronary spasm in patients with MINOCA undergoing provocative Ach testing. The authors found that myocardial bridging is present in almost 21% of MINOCA patients with vasomotion abnormalities and represents within this population an independent predictor of a worse outcome.

### Unmet Clinical Needs

While the clinical management of AMI with obstructive coronary artery disease (CAD) is guided by frequently updated evidence-based guidelines released by the international scientific society (28–31), no specific guidelines or treatment recommendations are currently available for MINOCA due to the paucity of dedicated clinical trials focused on this population. In addition, optimal medical therapy for these patients is not currently established due to the lack of dedicated trials evaluating clinical outcomes of commonly used medications for secondary prevention in MINOCA patients. However, an etiology-based tailored approach should be followed as a general recommendation.

## Invasive Functional Assessment: Why So Critical?

**Figure 1:**
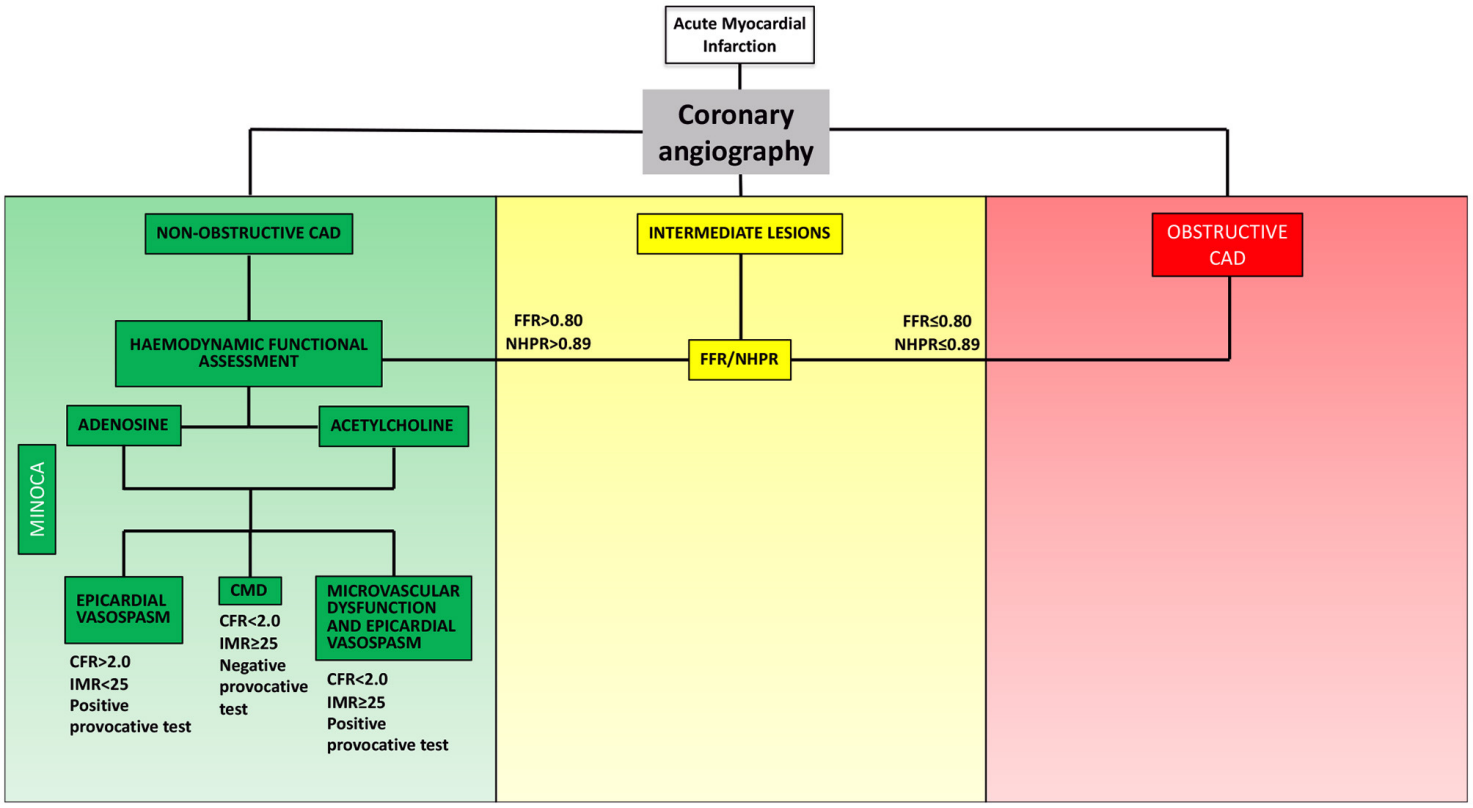
Invasive functional assessment of MINOCA. MINOCA, myocardial infarction with non-obstructive coronary arteries; CAD, coronary artery disease; FFR, fractional flow reserve; NHPR, non-hyperaemic pressure ratios; CFR, coronary flow reserve; IMR, index of microvascular resistance.

In order to establish a tailored therapeutic strategy, an invasive evaluation aiming to identify the pathophysiological mechanism underlying MINOCA plays a crucial role (Figure 1). Once ruling out atherosclerotic causes of MINOCA (i.e., acute plaque destabilization due to rupture or erosion) via coronary angiography and intracoronary imaging techniques, one or more of the following tests could be used in the invasive diagnostic work-up of MINOCA: (1) functional assessment of angiographically intermediate coronary stenosis with fractional flow reserve (FFR) or non-hyperaemic pressure ratios (NHPR) to rule out obstructive CAD; (2) thermodilution- or Doppler-based assessment of coronary microvascular function; (3) provocative test with acetylcholine/ergonovine to rule out coronary vasoreactivity.

Hemodynamic profiles of CMD and vasospasm emerging via invasive pressure-flow assessment are summarized in Table 2.

**Table 2:** Hemodynamic profiles of coronary microvascular dysfunction (CMD) and epicardial vasospasm.

**Pathophysiological mechanisms**	**Hemodynamic profiles**	
Coronary microvascular dysfunction • Structural remodeling	Adenosine test • CFR < 2.0 • IMR ≥ 25	Acetylcholine test • No ischemic symptoms and ECG changes • No or <90% coronary diameter reduction
Coronary microvascular dysfunction • Microvascular spasm	Adenosine test • CFR < 2.0 • IMR < 25	Acetylcholine test • Ischemic symptoms and ECG changes • No or <90% coronary diameter reduction
Coronary microvascular dysfunction • Structural remodeling and microvascular spasm	Adenosine test • CFR < 2.0 • IMR ≥ 25	Acetylcholine test • Ischemic symptoms and ECG changes • No or <90% coronary diameter reduction
Coronary epicardial vasospasm	Adenosine test • CFR < 2.0 • IMR < 25	Acetylcholine test • Ischemic symptoms and ECG changes • ≥90% coronary diameter reduction
Coronary microvascular dysfunction and epicardial vasospasm	Adenosine test • CFR < 2.0 • IMR ≥ 25	Acetylcholine test • Ischemic symptoms and ECG changes • ≥90% coronary diameter reduction

*CFR, coronary flow reserve; IMR, index of microvascular resistance*.

### Epicardial CAD: Hyperaemic and Non-hyperaemic Pressure Ratios

The absence of obstructive CAD at coronary angiography is a major criterium for MINOCA diagnosis, therefore the presence of angiographically intermediate coronary stenosis deserves a functional evaluation via hyperaemic or non-hyperaemic indexes.

Among hyperaemic tests, FFR (88% sensitivity, 100% specificity, 100% positive predictive value, 88% negative predictive value, and 93% accuracy (32), the ratio between mean distal pressure and mean aortic pressure during maximal hyperaemia ($\frac{Pdhyper}{Pahyper}$), is the most widely used and accepted index for a physiological evaluation of epicardial coronary disease. International guidelines recommend a specific cut-off (FFR ≤ 0.80) for the detection of functionally relevant CAD (33).

On the other hand, resting physiological indexes may be adopted in order to assess the functional significance of coronary lesions with no pharmacological administration of hyperaemic agents: at this regard, instantaneous wave-free ratio [iFR−73% sensitivity, 87.8% specificity, 77% positive predictive value, 85.3% negative predictive value (34)], the ratio between mean distal pressure and mean aortic pressure during the diastolic wave-free period ($\frac{Pdwave-free}{Pawave-free}$) (35), resulted sufficiently accurate when compared to FFR with the pre-specified cut-off of 0.89 (34) and, more importantly, non-inferior to FFR in terms of clinical outcomes in two large scale, randomized, clinical trials (36, 37).

Several other NHPR have been proposed for adenosine-free functional assessment of CAD: the resting full-cycle ratio (RFR–the lowest Pd/Pa within the entire cardiac cycle (38), diagnostic accuracy 97.4%, sensitivity 98.2%, specificity 96.9%, positive predictive value 94.5%, negative predictive value 99.0%) and diastolic pressure ratio (dPR–accuracy 87.5%, sensitivity 62.3%, specificity 95.6%, positive predictive value 82.0% and negative predictive value 88.7%) which is an averaged Pd/Pa ratio during a part or the entire diastolic period without selection of a wave-free period. These NHPR (pre-specified cut-off of 0.89) showed a significant correlation with iFR (39) and then emerged as reliable alternative tools to guide treatment strategy in patients with coronary artery disease (40, 41).

### Microvascular CAD: From Coronary Flow to Microvascular Resistance

A comprehensive coronary physiology assessment including the appraisal of both the epicardial and the microvascular compartments provides a detailed analysis of ischemic heart disease, particularly among patients with non-critical epicardial CAD who may benefit from a more accurate investigation of CMD in order to establish the pathophysiological mechanism underlying the ischemic symptoms. Although CFR cannot specifically assess the contribution of the microvasculature to ischemic heart disease, it is generally accepted that, in the absence of significant epicardial disease, an impairment of CFR reflects the presence of CMD. From a technical point of view, CFR may be measured invasively using Doppler flow velocity or thermodilution. The use of a Doppler flow-pressure wire allows the assessment of CFR as the ratio of hyperaemic [after 140 mg/kg/min of intravenous adenosine (42)] to resting coronary flow velocity (CFV): $\frac{CFVhyper}{CFV\;rest}$ (43). On the other hand, the use of a specific temperature-pressure wire enables CFR measurement through thermodilution as resting mean transit time (Tmn) divided by hyperaemic Tmn (CFR = $\frac{Tmn\;baseline}{Tmn\;hyperemic}$), showing a strong correlation with true coronary flow (44, 45). Cut-off values of <2.0 for thermodilution-based measurement (46, 47) and <2.5 for Doppler-based measurement (48, 49) showed the strongest prognostic impact (sensitivity 86–92%, specificity 89–100%, diagnostic accuracy 89–96%, positive predictive value 84–100%, negative predictive value 77–95%).

Microvascular function can be specifically estimated with the thermodilution-based index of microvascular resistance (IMR—sensitivity 64%, specificity 75% (50). According to Ohm's law, vascular resistance (R) is equal to driving pressure (ΔP) divided by flow rate (Q): *R* = ΔP/Q. ΔP is the pressure difference across the myocardium (Pd – Pv) and Q represents the coronary flow, which is known to be inversely related to Tmn ($\frac{1}{Tmn}$). Therefore, coronary microvascular resistance (R) can be calculated as follows: Pd–Pv/$\frac{1}{Tmn}$ = (Pd–Pv) × Tmn. Assuming that venous pressure is close to zero (Pv = 0), the final equation will be: Pd × Tmn. Therefore, the index of coronary microvascular resistance (IMR) is calculated with thermodilution as the product of distal coronary pressures (Pd) and Tmn during maximal hyperemia (IMR = Pd × Tmn_[*hyper*]_) (51). Of note, a strong correlation between IMR and true microcirculatory resistance (TMR) was found. In particular, IMR values ≥25 suggest high microvascular resistance and, therefore, CMD (52).

Both CFR and IMR are thermodilution-based physiological indexes in which coronary flow and microvascular resistances are indirectly estimated via the Tmn of a manually injected saline bolus, thus implying that its measurement depends to a certain degree on the injection technique. Moreover, both CFR and IMR require the achievement of adenosine-induced stable hyperemia. The measurement of absolute coronary flow (Q) and microvascular resistance (R) has been proposed to overcome these limitations by adopting a thermodilution technique that requires a continuous infusion of saline through a dedicated monorail catheter. Such equipment allows the infusion of saline at room temperature infused at a pre-specified flow rate (Qi, 20 mL/min for the left anterior descending [LAD] and left circumflex artery [LCx] and 15 mL/min for the right coronary artery [RCA]), resulting in a hyperaemic state similar to that produced by adenosine. The temperature of the infused saline (Ti) and of the saline/blood mixture (T), and the distal coronary pressure (Pd) are measured with a pressure/temperature sensor-tipped guide wire. Q is calculated as 1.08 x (Ti/T) x Qi, and expressed in ml/min. R is calculated as Pd/Q, and expressed as mm Hg/L/min, or Wood units. These measurements present several advantages over the traditional CFR and IMR, as they are safe, reproducible, and not operator-dependent. Moreover, they do not require pharmacological-induced hyperemia since continuous saline injection produces a prolonged and steady physiological hyperemic state within seconds.

The physiological finding of an impaired CFR in patients with ischemic heart disease and non-obstructive CAD currently meets a general agreement. Multiple contributions support the hypothesis that, even in the absence of critical epicardial CAD, microvascular dysfunction implies impairment of blood flow across coronary vessels (53), which may also reflect an abnormal resting coronary flow velocity, with a subsequent adverse myocardial performance, thus potentially leading to unfavorable prognosis (54, 55). Interestingly, in a cohort of patients with ischemia and non-obstructive coronary arteries (INOCA) who underwent invasive physiological assessment, also including FFR, acetylcholine testing, and adenosine administration, absolute coronary flow measured with continuous thermodilution, resulted as the best predictor of self-reported angina (56).

However, the body of evidence concerning coronary flow and flow reserve measurement among the MINOCA population is currently limited. Similarly, the role and the clinical implications of continuous thermodilution-derived indexes within MINOCA patients are not yet established. A recent contribution by Mochula et al. (57) showed that MINOCA patients develop a mild reduction of myocardial blood flow and perfusion as assessed by SPECT myocardial perfusion scintigraphy (MPS). Indeed, it provides a further proof that despite the absence of obstructive CAD this subset of patients has more pronounced risk of cardiac events needing of more aggressive observation and treatment. Yet, the prognostic impact of CFR impairment as well as the reference values to adequately establish it are still matter of debate.

A recent work by Konst et al. (56) reported that absolute microvascular resistance, as assessed by continuous thermodilution, was significantly increased in INOCA, thus suggesting that a functional impairment of the coronary microvascular district plays a central role in INOCA pathophysiological mechanisms.

According to the microvascular resistance status, several combinations of epicardial and microvascular disease are available (58). The prognostic impact of discordant coronary physiology indexes has been largely debated (58, 59). Focusing on ischemic heart disease with non-obstructive CAD, patients with preserved FFR (>0.80) but reduced CFR (<2) have been shown to experience a higher incidence of unfavorable outcomes compared with those with preserved FFR and CFR, thus highlighting the critical prognostic role of CMD in ischemia-driven adverse events (49).

On the other hand, contributions investigating the potential prognostic impact of CMD among MINOCA population are limited to exploratory results deriving from observational studies. In particular, a recent study by Abdu et al. is worth mentioning. Coronary microvascular function was assessed in a small cohort of 109 MINOCA patients with the coronary angiography-derived index of microvascular resistance (caIMR), a novel pressure-wire-free index of CMD evaluation. Interestingly, a caIMR value >43U has been reported in most of the patients and resulted a strong independent predictor of major adverse outcomes as well as a useful tool for risk stratification among MINOCA patients (60), although adequately powered randomized clinical trials specifically comparing the prognostic impact of both caIMR and traditional IMR within MINOCA population are warranted to address this issue. Similarly, there are limited evidence evaluating whether the cut-off values for both CFR and IMR in the acute setting of MINOCA are equivalent to those commonly established in patients with stable ischemic heart disease.

In addition, several non-invasive methods of coronary microvascular function assessment have been tested: particularly, a recent study by De Vita et al. enrolling a small cohort of MINOCA patients found an abnormal coronary flow velocity response to both ergonovine and adenosine as assessed by trans-thoracic Doppler echocardiography, thus indirectly suggesting a significant microvascular impairment within this population (61).

### Provocative Tests

Although current guidelines do not recommend provocative tests in patients with AMI (28, 29), several pieces of evidence support their adoption in the presence of a reasonable clinical suspect of coronary vasomotion abnormalities for epidemiological and prognostic reasons. In this regard, the epidemiological burden of coronary vasospasm among MINOCA patients has not yet been clarified. However, according to the most recent estimates, clinically relevant vasomotion abnormalities have been found in a large cohort of MINOCA patients undergoing acetylcholine provocative tests (~46%) (26).

In addition, although the impact of a positive provocative test is limited in patients presenting with unstable angina and non-obstructive coronary arteries (62, 63), the prognostic implications of a positive test in patients with AMI appear to be relevant since patients with MINOCA and underlying impaired coronary vasomotor tone show a more frequent occurrence of death for all causes, cardiac death and readmission for ACS as well as worse angina status (26). The latter evidence supports the hypothesis that a positive provocative test among the MINOCA population identifies a sub-group of patients at higher risk.

Multiple safety concerns have risen since invasive provocative tests showed a potential risk of malign ventricular arrhythmias as well as bradyarrhythmias development. However, several studies offer encouraging evidence showing that the overall incidence of arrhythmic complications is comparable with that occurring during a spontaneous anginal attack, thus suggesting that provocative tests do not provide significant additional arrhythmic risk (26, 64, 65).

## Etiology-Tailored Therapeutic Strategies

The recent trend toward tailored therapeutic management guided by a comprehensive invasive evaluation of the pathophysiological mechanisms of ischemia in patients with non-obstructive coronary arteries has been set by the British Heart Foundation Coronary Microvascular Angina (CorMicA) trial (66). In this pivotal study, the authors demonstrated that a strategy of adjunctive invasive assessments of coronary function followed by a stratified and etiology-based therapy led to a reduction in angina severity and improved quality of life. In particular, consecutive patients with angina in the absence of angiographically relevant obstructive CAD were randomized 1:1 to the interventional group (invasive functional evaluation with tailored stratified therapeutic strategy) or control group (standard care). At 1 year of follow-up, the intervention significantly improved angina and quality of life, even without relevant differences in MACE (67).

Although antiplatelet therapy represents one of the cornerstones of secondary prevention of AMI with obstructive coronary arteries, considerable evidence has suggested a neutral (68) or even detrimental (69) effect of antiplatelet therapy among the overall heterogeneous cohort of MINOCA patients, further highlighting that the management strategies should be strictly based on the findings arising from a careful invasive evaluation. Selectively focusing on patients with coronary plaque disruption (i.e., plaque rupture or erosion), there is unequivocal evidence of a benefit from lifetime single antiplatelet therapy with aspirin. Moreover, 1 year of dual antiplatelet therapy with the addition of a P_2_Y_12_ receptor inhibitor should be considered in these patients in light of the significant role of thrombosis and distal embolization in the pathogenesis of MINOCA with plaque disruption (12).

The optimal management of SCAD is still a matter of debate since no randomized clinical trials have compared medical therapy to revascularization strategies. However, according to the available data, except for type-1 SCAD obstructing coronary flow or hemodynamically unstable patients presenting with STEMI, a conservative approach aiming at limiting the risk of dissection propagation following percutaneous coronary interventions (PCI) is associated with acceptable outcomes and should therefore be preferred (70–73). Hypertension is a well-known independent predictor of recurrent SCAD (74), and strict blood pressure control with ACE/ARBs and/or beta-blockers associated with single antiplatelet therapy is the mainstay of conservative SCAD management. In contrast, dual antiplatelet therapy should be reserved for patients undergoing PCI and stent implantation.

In patients with evidence of either epicardial or microvascular spasm following provocative tests, calcium channel blockers (dihydropyridine, non-dihydropyridine, or both) should be considered first-line therapy due to their ability to induce smooth muscle cell relaxation and decrease myocardial oxygen consumption (75). Conversely, in patients with CMD due to arteriolar remodeling and capillary rarefaction presenting with reduced CFR, increased IMR/HMR, and negative acetylcholine test, ACE-I and beta-blockers seem a more reasonable option (66, 76, 77).

Lastly, when coronary thromboembolism is suspected, the standard treatment is strictly related to the cause of embolism. According to dedicated guidelines, long-term or even lifetime anticoagulation therapy may be suggested in patients affected by acquired/inherited thrombophilia. In patients with paradoxical embolism due to ASD, transcatheter device closure or surgical repair is recommended (78), whereas secondary prevention of PFO-related embolism include the administration of long-term antiplatelet therapy or trans-catheter device closure (79, 80).

## Conclusion

MINOCA is a complex clinical syndrome with a broad spectrum of pathophysiological mechanisms, among which vasomotion abnormalities and coronary microvascular dysfunction (CMD) play a significant role. Invasive functional assessment has a limited body of evidence in the acute setting, and therefore current guidelines do not recommend routine physiological investigation of epicardial and microvascular coronary compartments within the context of AMI. Furthermore, several invasive tests may indirectly alter microvascular function and status: for example, the pharmacological stimuli with acetylcholine conventionally used to evaluate epicardial vasomotion abnormalities may also have constrictor effects on smooth muscle cells at the microvascular level, thus contributing to an impaired microvascular response. Therefore, it is not always possible to attribute CMD to a specific mechanism since it may frequently recognize multiple underlying causes potentially overlapping. However, despite all these limitation, several studies suggest that a comprehensive invasive functional assessment may help identify the underlying pathophysiological mechanism of myocardial ischemia and consequently select an appropriate therapeutic strategy. Although current guidelines do not recommend routine physiological assessment of epicardial and microvascular coronary compartments within the context of AMI, several pieces of evidence support the pivotal role of a comprehensive invasive functional assessment to identify the underlying pathophysiological mechanism and consequently to select an appropriate therapeutic strategy. Unfortunately, the optimal medical therapy for these patients is not currently established; however, a tailored etiology-based approach should be followed as a general recommendation. Lastly, there is an urgent need for randomized trials to evaluate the short and long-term effects of secondary prevention measures and etiology-targeted therapies to improve the management and prognosis of this heterogeneous population.

## Author Contributions

FM and MV wrote the first draft of the manuscript. All authors contributed to manuscript revision, read, and approved the submitted version.

## Conflict of Interest

The authors declare that the research was conducted in the absence of any commercial or financial relationships that could be construed as a potential conflict of interest.

## Publisher's Note

All claims expressed in this article are solely those of the authors and do not necessarily represent those of their affiliated organizations, or those of the publisher, the editors and the reviewers. Any product that may be evaluated in this article, or claim that may be made by its manufacturer, is not guaranteed or endorsed by the publisher.
